# Histone acetyltransferase P300 deficiency promotes ferroptosis of vascular smooth muscle cells by activating the HIF-1α/HMOX1 axis

**DOI:** 10.1186/s10020-023-00694-7

**Published:** 2023-07-06

**Authors:** Juan Shi, Qun-Hui Wang, Xiang Wei, Bo Huo, Jian-Nan Ye, Xin Yi, Xin Feng, Ze-Min Fang, Ding-Sheng Jiang, Ming-Jia Ma

**Affiliations:** 1 Division of Cardiothoracic and Vascular Surgery, Tongji Hospital, Tongji Medical College, Huazhong University of Science and Technology, 1095 Jiefang Ave, 430030 Wuhan, Hubei China; 2 Key Laboratory of Organ Transplantation, Ministry of Education; NHC Key Laboratory of Organ Transplantation; Key Laboratory of Organ Transplantation, Chinese Academy of Medical Sciences, Wuhan, Hubei China; 3 Department of Cardiology, Renmin Hospital of Wuhan University, Wuhan, China

**Keywords:** Vascular smooth muscle cells, Ferroptosis, P300, HIF-1α, HMOX1, P53

## Abstract

**Background:**

E1A-associated 300-kDa protein (P300), an endogenous histone acetyltransferase, contributes to modifications of the chromatin landscape of genes involved in multiple cardiovascular diseases. Ferroptosis of vascular smooth muscle cells (VSMCs) is a novel pathological mechanism of aortic dissection. However, whether P300 regulates VSMC ferroptosis remains unknown.

**Methods:**

Cystine deprivation (CD) and imidazole ketone erastin (IKE) were used to induce VSMC ferroptosis. Two different knockdown plasmids targeting P300 and A-485 (a specific inhibitor of P300) were used to investigate the function of P300 in the ferroptosis of human aortic smooth muscle cells (HASMCs). Cell counting kit-8, lactate dehydrogenase and flow cytometry with propidium iodide staining were performed to assess the cell viability and death under the treatment of CD and IKE. BODIPY-C11 assay, immunofluorescence staining of 4-hydroxynonenal and malondialdehyde assay were conducted to detect the level of lipid peroxidation. Furthermore, co-immunoprecipitation was utilized to explore the interaction between P300 and HIF-1α, HIF-1α and P53.

**Results:**

Compared with normal control, the protein level of P300 was significantly decreased in HASMCs treated with CD and IKE, which was largely nullified by the ferroptosis inhibitor ferrostatin-1 but not by the autophagy inhibitor or apoptosis inhibitor. Knockdown of P300 by short-hairpin RNA or inhibition of P300 activity by A-485 promoted CD- and IKE-induced HASMC ferroptosis, as evidenced by a reduction in cell viability and aggravation of lipid peroxidation of HASMCs. Furthermore, we found that hypoxia-inducible factor-1α (HIF-1α)/heme oxygenase 1 (HMOX1) pathway was responsible for the impacts of P300 on ferroptosis of HASMCs. The results of co-immunoprecipitation demonstrated that P300 and P53 competitively bound HIF-1α to regulate the expression of HMOX1. Under normal conditions, P300 interacted with HIF-1α to inhibit HMOX1 expression, while reduced expression of P300 induced by ferroptosis inducers would favor HIF-1α binding to P53 to trigger HMOX1 overexpression. Furthermore, the aggravated effects of P300 knockdown on HASMC ferroptosis were largely nullified by HIF-1α knockdown or the HIF-1α inhibitor BAY87-2243.

**Conclusion:**

Thus, our results revealed that P300 deficiency or inactivation facilitated CD- and IKE-induced VSMC ferroptosis by activating the HIF-1α/HMOX1 axis, which may contribute to the development of diseases related to VSMC ferroptosis.

**Supplementary Information:**

The online version contains supplementary material available at 10.1186/s10020-023-00694-7.

## Introduction

Ferroptosis, a nonapoptotic form of regulated cell death, is driven by iron-dependent lipid peroxidation (Wei et al. 2020). In recent years, tremendous efforts have been made to elucidate the effects of ferroptosis on the development of cardiovascular diseases, including aortic dissection, doxorubicin-induced cardiotoxicity, ischemia/reperfusion induced cardiomyopathy, heart failure and stroke (Chen et al. 2022a; Li et al. 2022; Yang et al., 2023). In our latest studies, we found that the protein levels of TFR, HOMX1 and ferritin, which are well-known regulators of cellular iron hemostasis, were significantly increased in the aortas of Stanford type A aortic dissection (TAAD) patients (Chen et al. 2022b). Additionally, in another study, we found that key regulators of SLC7A11, FSP1, and GPX4 in ferroptosis were significantly downregulated in aortas of TAAD patients (Li et al. 2022). Liao et al. also reported that the level of lipid peroxidation in aortic media homogenates was significantly higher in the thoracic aortic dissection group than in the normal group (Liao et al. 2008). These evidences indicated that ferroptosis played an important role in medial VSMC loss and the development of aortic dissection. Thus, further research on the regulatory mechanisms of VSMC ferroptosis will be beneficial for elucidating the pathological mechanisms of aortic dissection.

Acetylation, a posttranslational modification, epigenetically regulates gene expression and gene transcriptional activity (Peleg et al. 2016). It participates in the onset of various cardiovascular diseases, including obesity, diabetes mellitus, cardiometabolic diseases, ischemia‒reperfusion injury, cardiac remodeling, hypertension, and arrhythmias (Yang et al. 2020a, b). Our previous study demonstrated that histone acetylation was involved in the pathogenesis of aortic dissection; for example, the levels of H4K12ac and H3K23ac were increased, while H3K18ac, H4K8ac and H4K5ac were decreased in the aortas of TAAD patients (Guo et al. 2019). Although it has been reported that class I histone deacetylase inhibitors selectively protect neurons but augment ferroptosis in cancer cells (Zille et al. 2019), whether acetylation participates in ferroptosis in VSMCs is unknown.

E1A-associated 300-kDa protein (P300), a classic endogenous histone acetyltransferase, is related to several cardiovascular pathologies including vascular dysfunction, cardiac hypertrophy, myocardial infarction, cardiac fibrosis, systolic/diastolic dysfunction, and aortic valve calcification (Bouchal et al. 2011; Rai et al. 2017). Recently, P300 was reported to inhibit arterial injury-induced intimal hyperplasia by promoting contractile protein expression and inhibiting migration of VSMCs (Chakraborty et al. 2022). The protein level of P300 was reported to be significantly elevated in human atherosclerotic samples and contributed to oxidative stress in atherogenesis by enhancing nicotinamide adenine dinucleotide phosphate (NADPH) oxidase 5 gene promoter activity, which is a major contributor to oxidative stress during atherosclerotic plaque development (Vlad et al. 2019). Recently, a few studies demonstrated that P300 was associated with the regulation of reactive oxygen species (ROS) production. For example, Zhou et al. found that iron overload in macrophages induced ROS production and enhanced the activity of P300/CBP acetyltransferase to polarize macrophages to the M1 subtype, which further enhanced the antitumor effect of tumor-infiltrating macrophages (Zhou et al. 2018). ROS production and inflammation are considered drivers of ferroptosis (Chen et al. 2022b; Wei et al. 2020), but whether P300 plays a critical role in VSMC ferroptosis remains unclear.

In the present study, we found that the expression of P300 was significantly decreased in HASMCs treated with cystine deprivation (CD) and imidazole ketone erastin (IKE) to induce ferroptosis, and this decrease in P300 expression was restored by ferrostatin-1 (Fer-1) treatment. Furthermore, we demonstrated that P300 deficiency or inhibition promotes CD- and IKE-induced ferroptosis of VSMCs by activating the HIF-1α/HMOX1 axis. Therefore, our findings reveal a novel regulatory mechanism of ferroptosis and provide potential targets for the treatment of aortic dissection.

## Methods and materials

### Cells isolation and culture

Primary human aortic smooth muscle cells (HASMCs) were harvested from healthy ascending aortas of donors during heart transplantation surgeries as previously described (Chen et al. 2020). This study was approved by the Tongji Hospital, Tongji Medical College, Huazhong University of Science and Technology Review Board. The aortic wall was transferred to the laboratory in prechilled DME/F12 medium (SH30023.01, HyClone) as soon as the tissue was isolated from the donors. After carefully peeling off the intima and adventitia of the aorta, the media was torn into 3–5 layers with micro tweezers in cold DME/F12 medium. Then, the peeled and thin media of the aorta was transferred into a culture flask and cut into 1 mm tissue blocks, which were spread evenly on the bottom of the flask with scissors. The flask was subsequently placed in the incubator and stood upright for 30–40 min. After making sure the tissue blocks attached to the wall of the flask, 7 mL DME/F12 medium containing 10% fetal bovine serum (SH30084.03, Hyclone) and 1% penicillin-streptomycin (15140-122, Thermo Fisher Scientific) was add into the culture flask. After 1 week, some spindle-shaped smooth muscle cells could be observed around the tissue pieces. When the HASMCs grew to a suitable density, they were digested with trypsin and transferred to ordinary culture dishes for experiments and passaged every 2–3 days.

### Plasmids construction

The knockdown plasmids for silencing targets were constructed as described previously (Li et al. 2018). Double-strand oligonucleotides of shRNA targeting human P300 and HIF-1α were inserted into the PLKO.1 vector, at the Age I and the EcoR I restriction enzyme sites. The target sequences are as below: human-shP300-1: 5’-gccttcacaattccgagacat-3’; human-shP300-2: 5’-cagacaagtcttggcatggta-3’; human-shHIF-1α: 5’-ccgctggagacacaatcatat-3’. The full-length human HIF-1α CDSs were amplified by PCR and cloned into the pHAGE lentiviral vector. HIF-1α forward primer: 5’-CGACGCGTCGATGAGCTCCCAATGTCGGAG-3’, HIF-1α reverse primer: 5’-GCTCTAGAGCAGAGCTTTGGATCAAGTTAAC-3’. The pEnCMV-P300-HA plasmid (P23868) was purchased from MiaoLing Plasmid Platform.

### Drug treatment and lentivirus infection

Lentivirus production and infection were performed as previous studies described (Chen et al. 2022b). The knockdown plasmids together with packaging plasmids psPAX2 (12260, Addgene) and pMD2.G (12259, Addgene) were co-transfected into 293T cells through polyethyleneimine (764604, Sigma-Aldrich), and the medium was changed 8 h after transfection. The supernatant medium containing virus was harvested at 24 and 48 h after medium change. When HASMCs grew to the appropriate density, they were infected with lenti-shRNA, lenti-shP300-1, lenti-shP300-2, or lenti-shHIF-1α for 24 h. After starvation for 8 h, the HASMCs infected by lentivirus were passaged into different culture dishes for subsequent experiments. 2.5 µM of Imidazole ketone erastin (IKE, S8877, Selleck) or cystine deprivation medium (CD, DZPYG0257, Boster Biological Technology) were used to induce ferroptosis and 2.5 µM of ferrostatin-1 (Fer-1, S7243, Selleck) was used to reverse ferroptosis. 3-MA (S2767, Selleck) and Emricasan (S7775, Selleck) were used at the concentration of 5 mM and 5 µM, respectively. A-485 (S8740, Selleck), an inhibitor of P300, was administered at the same time of IKE or CD. The inhibitor of HIF-1α, BAY87-2243 (S7309, Selleck), was used at a concentration of 20 µM. MG132 (HY13259, MedChemExpress) was used at a concentration of 3 µM.

### Protein extractions and western blot

Total proteins of HASMCs were extracted with radioimmunoprecipitation assay (RIPA) (20101ES60, YEASEN Biotech) solution containing phosphatase inhibitors (20109ES20, YEASEN Biotech) and protease inhibitors (20124ES10, YEASEN Biotech) as described previously (Jiang et al. 2016, 2017). The steps of nuclear protein extraction were as follow: HASMCs with indicated treatments were washed and collected in ice-cold PBS and pelleted. After incubation in cytoplasmic lysis buffer I (20 mM HEPES, 1 mM EDTA, 10 mM KCl, 2 mM MgCl_2_, 0.25% NP40 and protease inhibitors), the cells were lysed by vortex for 15 s and rotation for 30 min. Then, the pelleted nucleus were obtained after centrifugation for 5 min at 5000×g. The pelleted nucleus was lysed with nuclear lysis buffer II (20 mM HEPES, 1 mM EDTA, 420 mM NaCl, 10 mM KCl, 2 mM MgCl_2_, 0.25% NP40, 25% glycerol and protease inhibitors). After vortex for 15 s and rotation for 10 min, nuclear lysate was sonicated and centrifuged at 14,000×g for 5 min. Subsequently, the supernatant was collected as the nuclear lysate. After quantification using a BCA kit (23,227, Thermo Fisher Scientific), the proteins were separated by 8%, 10% or 12% sodium dodecyl sulfate-polyacrylamide gel electrophoresis (SDS-PAGE) and transferred to polyvinylidene fluoride (PVDF, IPVH00010, Millipore) membranes for 90 min or 3 h at 200 mA. Next, the membranes were blocked with 5% skim milk for 1 h at room temperature, and after washing with tris buffered saline tween (TBST) for three times, the membranes were incubated with corresponding the primary antibodies overnight at 4 °C. Then, the HRP-linked secondary antibody was used for secondary incubation for 2 h at room temperature after washing with TBST. Finally, the membranes were visualized by incubating with enhanced chemiluminescence reagents and exposed using the ChemiDocTM XRS + system (Bio-Rad). Primary antibodies used were: P300 (sc-48,343, Santa Cruz), HIF-1α (20960-1-AP, Proteintech Group), H3K18ac (ab40888, Abcam), H3K27ac (GTX128944, GeneTex), H2BK5ac (12799, Cell Signaling Technology), AIFM2/FSP1 (HPA042309, Atlas Antibodies; 20886-1-AP, Proteintech Group), GPX4 (ab125066, Abcam), β-actin (8457, Cell Signaling Technology), SLC7A11 (NB300-318, Novus Biologicals), P53 (A5804, ABclonal Technology), Lamin B1 (12987-1-AP, Proteintech Group) and GAPDH (AC033, ABclonal Technology).

### Cell viability assay

Cell viability was assessed by using the Cell Counting Kit-8 (CCK-8, BS350A, Biosharp) according to the manufacturer’s instructions. Briefly, HASMCs were seeded in 96-well plates at a density of 8000 cells each well in 100 µL culture medium. After adherent culture, the cells were treated with IKE or CD to induce ferroptosis for the indicated times. Then, 10 µL of the CCK8 reagent was added to 100 µL of medium in each well and the 96-well plate was incubated at 37 °C for 2 h. The plate was analyzed by measuring the optical density (OD) value at 450 nm using a microplate spectrophotometer (ELx808, BioTek, Winooski, VT). Similarly, a cytotoxicity lactate dehydrogenase (LDH) assay kit (CK12; Dojindo) was used to evaluate HASMCs injury under the different treatments. All procedures were performed in accordance with the manufacturer’s instructions and the OD value was measured at 490 nm. The proportion of viable cells in each group was present as normalized against that of the control wells. The levels of LDH in each well were calculated depending on the blank, high control and low control values.

### Flow cytometry with propidium iodide (PI) staining

PI staining (P4170, Sigma-Aldrich) were used to detect cell death. After HASMCs were treated with IKE or CD in 6-wells plate for the indicated times, the cells were collected. First, all the cells were digested with trypsin and collected together. The mixture was centrifuged at 1000 rpm for 5 min. Then, the supernatant was wiped off and the cells were resuspended in 200 µL binding buffer. Before flow cytometry was performed, PI dyes were added into the cell suspension at a final concentration of 5 µg/mL in the dark place for 15 min. The dyes could enter dead cells with damaged membranes and bind to nucleic acids, producing bright red fluorescence and the fluorescence intensity represents the degree of cell death.

### Malondialdehyde (MDA) assay

The relative MDA concentrations in HASMCs were assessed with a lipid peroxidation MDA assay kit (S0131M, Beyotime) according to the manufacturer’s instructions. HASMCs were seeded in 6-wells plates and cells in each well were lysed with 100 µL RIPA lysis buffer after treatment with CD, and IKE for the indicated times. After sonication and centrifugation, the supernatant was obtained. A BCA protein assay was used for total protein quantification. Next, 200 µL MDA working solution, which was composed of thiobarbituric acid (TBA) diluent, TBA storage solution and antioxidants, was added to 100 µL supernatant. After incubating at 100 °C for 15 min, the mixture was cooled to room temperature in water, and centrifuged (1000×g, 10 min) to remove insoluble material. Then, 100 µL of the supernatant was took out to determine the absorbance at 532 nm.

### Lipid peroxidation assay with BODIPY-C11

The BODIPY 581/591 C11 kit (D3861, Thermo Fisher Scientific) was used to measure lipid peroxidation levels. After treatment with IKE or CD for the indicated times, the cells were incubated with the BODIPY-C11 probe, which was added to medium at a final concentration of 5 µM for 30 min at 37 °C. Next, the cells were digested with trypsin, collected and centrifuged at 1000 rpm for 5 min. Then, the cells were resuspended in 300 µL PBS and transferred into tubes. Finally, cell fluorescence was acquired on a CytoFLEX-3 cytometer (Beckman Coulter). Oxidation of the dye causes the fluorescence emission peak to shift from 590 nm to 510 nm. The BODIPY-C11 value was calculated as the ratio of the oxidized probe to reduced probe fluorescence.

### Immunofluorescence staining

The HASMCs were seeded into 12-wells plates covered by round cell coverslips and treated with IKE or CD for the indicated times. The HASMCs were fixed with 4% polyformaldehyde for 20 min. After washing with PBS 3 times, the cells were permeabilized in 0.2% Triton X-100 for 20 min. Next, the cells were blocked in PBS containing 1% BSA for 1 h and incubated with the primary antibody, 4-hydroxynonenal (4-HNE, MAB3249, R&D Systems), overnight at 4 °C. Then, the secondary antibody was incubated for 2 h, and DAPI was incubated for 5 min in the dark. Fluorescence images of cells were collected under a fluorescence microscope. Exposure conditions in the same channel of different groups in each experiment were consistent.

### Co-immunoprecipitation assay

Co-Immunoprecipitation (Co-IP) assay were used to analyze the interaction between P300 and HIF-1α, and between P53 and HIF-1α. The interaction between P300 and HIF-1α was detected in total protein of HASMCs and 293T of three 10-centimeter culture dishes. After treatment with 3 µM MG132 for 12 h, the cells were lysed with 1 ml ice-cold hypotonic NP40 lysis buffer containing phosphatase and protease inhibitor. After sonicating, the samples were frozen in liquid nitrogen and thawed in water at room temperature 3 times. Then, the mixture was centrifuged at 12,000 rpm at 4 °C for 15 min, and 100 µL of the supernatant was reserved as input. The rest of the supernatant was collected, incubated with 4 µg of the indicated antibody (P300/HIF-1α) and rotated at 4 °C overnight. After nuclear protein extraction, the remaining steps were carried out following the same protocol as described above. Next day, after washing the magnetic beads (B23202, Biomake), 30 µL beads were added to each sample and incubated with the mixture for 3 h at 4 °C. Magnetic beads were collected by utilizing a magnetic stand and washed with 0.5 M NaCl in NP40 lysis buffer 4 times. After that, 70 µL of 1× SDS loading buffer was added to each sample and the samples were denatured at 95 °C for 30 min. The interaction among P300, HIF-1α and P53 was detected in nuclear protein extraction of HASMCs. HASMCs in eight 10 cm culture dishes were treated with CD to induce ferroptosis, and another eight untreated dishes were used as the control group. MG132 at a concentration of 3 µM was used in all dishes. After nuclear protein extraction, the remaining steps were carried out following the same protocol as described above. Finally, western blot was conducted to evaluate whether the immunoprecipitated proteins were pulled down.

### Statistical analysis

The data were analyzed by GraphPad Prism 9 software in this study. All the results were represented as mean ± standard deviation (SD). Multiple group comparisons were performed by using one-way ANOVA with post hoc analysis. Tukey’s multiple comparisons tests were used for multiple comparisons after one-way ANOVA. *p* value less than 0.05 is considered to be statistically significant.

## Results

### P300 was downregulated during ferroptosis of HASMCs

**Fig. 1:**
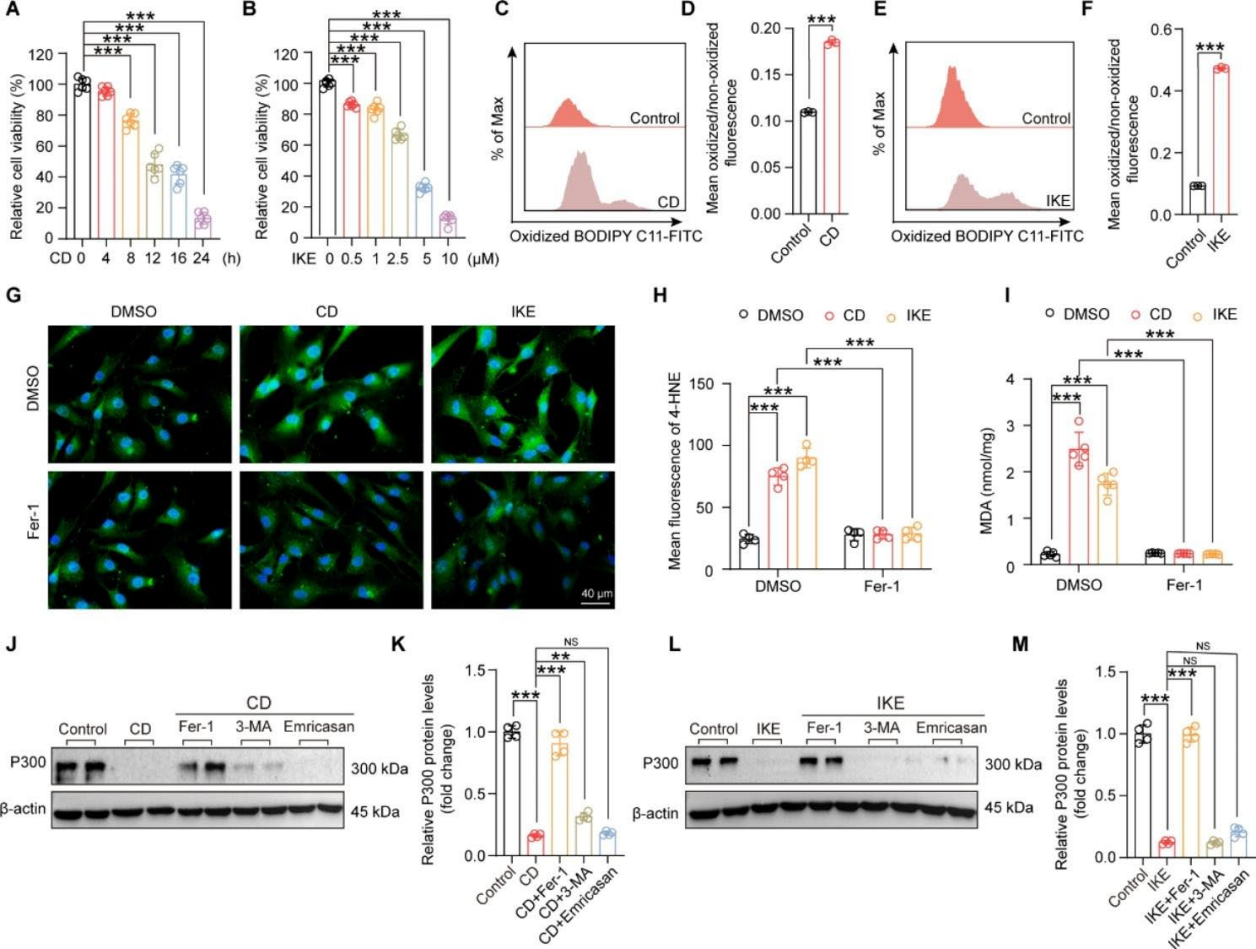
P300 was downregulated during ferroptosis of HASMCs. **(A)** Relative cell viability of HASMCs measured with a CCK8 kit after cystine deprivation (CD) treatment for 0, 4, 8, 12, 16, and 24 h (n = 6 per group). **(B)** Relative cell viability of HASMCs after treatment with different concentrations of imidazole ketone-erastin (IKE) (0, 0.5, 1, 2.5, 5, 10 µM) (n = 6 per group). **C-F.** The level of lipid ROS (oxidized BODIPY-C11/non-oxidized BODIPY-C11 ratio) detected by using a BODIPY-C11 kit in HASMCs after treatment with CD (C-D) and IKE (E-F) (n = 3 per group). **G.** Representative images of immunofluorescence staining with 4-HNE in HASMCs treated with DMSO and Fer-1 after CD and IKE stimulation for 16 h. **H.** Quantitative analysis of immunofluorescence staining of 4-HNE (n = 4 per group). **I.** The level of MDA measured by using an MDA assay kit in HASMCs treated with DMSO and Fer-1 after CD and IKE stimulation for 16 h. (n = 5 per group). **J-M.** P300 protein levels in HAMSCs after CD (J-K), IKE (L-M) stimulation for 16 h and treatment with Fer-1, 3-MA and Emricasan were measured by western blot (n = 4 per group). J and L. Representative western blot; K and M. Quantitative results. β-actin served as a loading control. Values are means ± SD; ***p < 0.001, **p < 0.01, NS, no significant

To investigate whether P300 is involved in HASMC ferroptosis, the classic ferroptosis cell models were first established by treating HASMCs with CD and IKE. The result of the CCK-8 assay showed that the viability of HASMCs was decreased in a time-dependent manner after treatment with CD for 0, 4, 8, 12, 16, and 24 h, and approximately 50% of the cells died at 16 h **(**Fig. 1A**)**. Similarly, the viability of HASMCs was found to decrease in a dose-dependent manner after treatment with different concentrations (0, 0.5, 1, 2.5, 5, and 10 µM) of IKE, and 2.5 µM IKE could induce ferroptosis in approximately half of the cells **(**Fig. 1B**)**. As lipid peroxidation is the hallmark of ferroptosis, we further evaluated the success of CD or IKE-induced ferroptosis by detecting lipid peroxidation using BODIPY-C11, immunofluorescence staining of 4-HNE and MDA assay. Our results demonstrated that both CD and IKE treatment significantly increased lipid peroxidation in HASMCs (Fig.1C-I), and these effects were almost completely reversed by the ferroptosis inhibitor Fer-1 **(**Fig. 1 G-I**)**. Thus, these results indicated that CD and IKE effectively induced ferroptosis of HASMCs.

To clarify whether P300 contributes to ferroptosis of HASMCs, we further measured the protein level of P300. Our results revealed that in normal HASMCs, P300 was highly expressed, while CD or IKE treatment significantly reduced P300 expression levels **(**Fig. 1J-M**)**. More importantly, the decrease in P300 expression caused by CD or IKE treatment was largely reversed by the ferroptosis inhibitor Fer-1 but not by the autophagy inhibitor 3-methyladenine (3-MA) or the apoptosis inhibitor Emricasan **(**Fig. 1J-M**)**. These results suggested that the CD- or IKE-induced decrease in P300 expression is ferroptosis dependent.

### Knockdown of P300 promoted ferroptosis of HASMCs

**Fig. 2:**
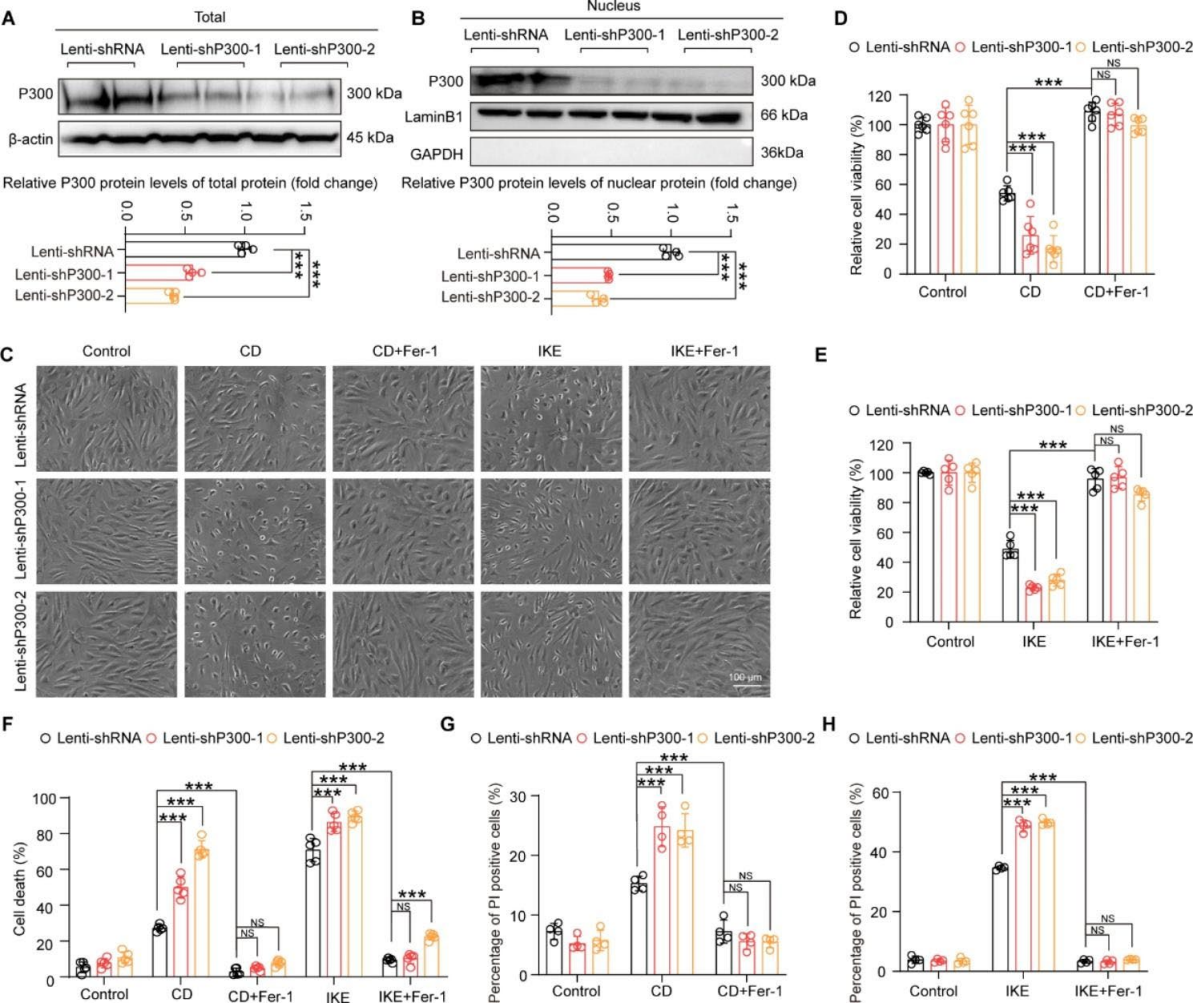
Knockdown of P300 reduced cell viability and increased cell death of HASMCs with CD- and IKE-induced ferroptosis. **A-B.** The protein level of P300 were detected by western blot in total and nuclear protein extraction of HASMCs infected with lenti-shRNA or lenti-shP300. (n = 4 per group). **(A)** Total protein extraction western blot; **(B)** Nuclear protein extraction. **C.** Representative images showing cell death induced by CD, IKE, alone or in combination with Fer-1 after HASMCs were infected with lenti-shRNA, lenti-shP300-1 and lenti-shP300-2. **D-E.** Relative viability of the indicated lentivirus-infected HASMCs evaluated by CCK8 after treatment with CD **(D)**, IKE **(E)**, alone or in combination with Fer-1 (n = 6 per group). **F.** Relative cell death of HASMCs infected with the indicated lentivirus evaluated by LDH after the treatment described above (n = 5 per group). **G-H.** Percentage of PI positive indicated lentivirus-infected HASMCs examined by flow cytometry with propidium iodide (PI) staining after treatment as described above (n = 4 per group). Values are means ± SD; ***p < 0.001, NS, no significant

To verify the role of P300 in HASMC ferroptosis, we first generated two different short-hairpin RNA (shRNA) plasmids to knockdown P300 (shP300-1and shP300-2) and considering the nature of the P300 transcriptional co-activator acting in the nucleus, we tested the total and nuclear protein expression level of P300 in HASMCs after infection with indicated lentiviruses. The results showed that both of shRNAs targeting P300 dramatically decreased the protein level of P300, as observed in both total and nuclear protein extraction (Fig. 2A and B**).** Then, HASMCs infected with the indicated lentiviruses were treated with CD or IKE to induce ferroptosis. From the cell images under the light microscope, we found that both CD and IKE significantly promoted cell death, and P300 knockdown further facilitated CD- and IKE-induced ferroptosis of HASMCs **(**Fig. 2C**)**. The results of the CCK-8 assay showed that compared with the control, there was an approximately 50% reduction in cell viability after CD and IKE stimulation, which was aggravated by P300 knockdown **(**Fig. 2D and E**)**. Furthermore, the LDH assay and PI staining were performed to evaluate cell injury and death. Consistent with the above results, P300 deficiency markedly accelerated cell injury and death induced by CD and IKE **(**Fig. 2F-H**)**. More importantly, the ferroptosis inhibitor Fer-1 largely reversed CD- and IKE-induced ferroptosis, and the detrimental effects of P300 deficiency in ferroptosis of HASMCs **(**Fig. 2C-H**)**. These results revealed that knockdown of P300 accelerated the ferroptosis of HASMCs induced by CD and IKE treatment.

**Fig. 3:**
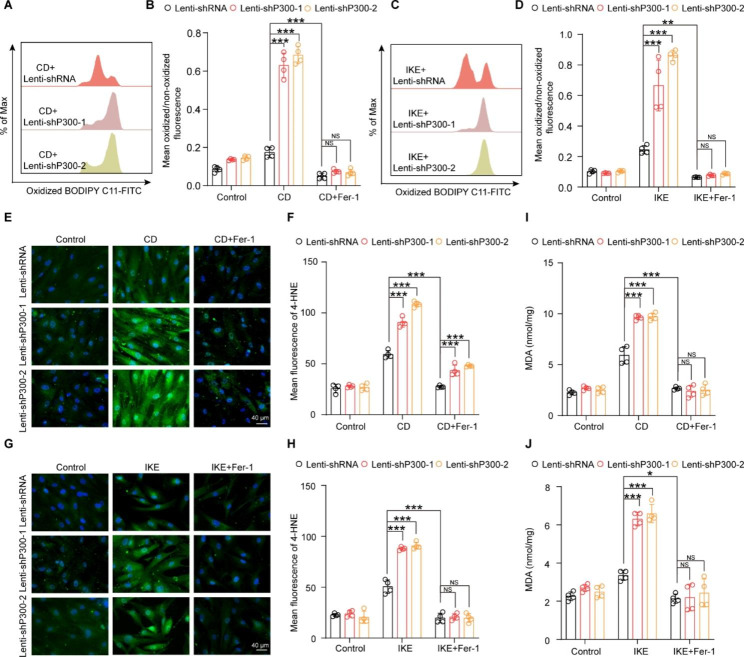
Downregulation of P300 aggravated the accumulation of lipid peroxidation during HASMC ferroptosis. **A-D.** The level of lipid ROS (oxidized BODIPY-C11/non-oxidized BODIPY-C11 fluorescence ratio) examined by using BODIPY-C11 kit after treatment with CD (A-B) and IKE (C-D), alone or in combination with Fer-1, in HASMCs infected with lenti-shRNA, lenti-shP300-1 and lenti-shP300-2 (n = 4 per group). **E and G.** Representative images of immunofluorescence staining of 4-HNE in the indicated lentivirus-infected HASMCs treated with CD (E), IKE (G), alone or in combination with Fer-1. **F and H.** Quantitative analysis of 4-HNE (n = 4 per group). **I-J.** The level of MDA was evaluated by an MDA assay kit in indicated lentivirus-infected HASMCs after CD (I), IKE (J), or in combination with Fer-1 stimulation for the indicated time (n = 4 per group). Values are means ± SD; ***p < 0.001, **p < 0.01, *p < 0.05, NS, no significant

Considering that ferroptosis is a type of regulated cell death triggered by lipid peroxidation, we further investigated whether P300 could regulate this biological process. As reported, both CD and IKE strikingly boosted lipid peroxidation, as evidenced by the increased ratio of oxidized to non-oxidized BODIPY-C11 **(**Fig. 3A-D**)**, 4-HNE levels **(**Fig. 3E-H**)** and MDA contents **(**Fig. 3I-J**)** of HASMCs. Moreover, the results of the lipid peroxidation BODIPY-C11 assay, 4-HNE immunofluorescence staining and MDA assay demonstrated that knockdown of P300 significantly increased lipid peroxidation induced by CD and IKE of HASMCs **(**Fig. 3A-J**)**. In addition, Fer-1 abolished the impacts of CD, IKE and their synergistic effects with P300 knockdown on lipid peroxidation of HASMCs **(**Fig. 3A-J**)**.

### The function of P300 in ferroptosis is acetyltransferase activity-dependent in HASMCs

**Fig. 4:**
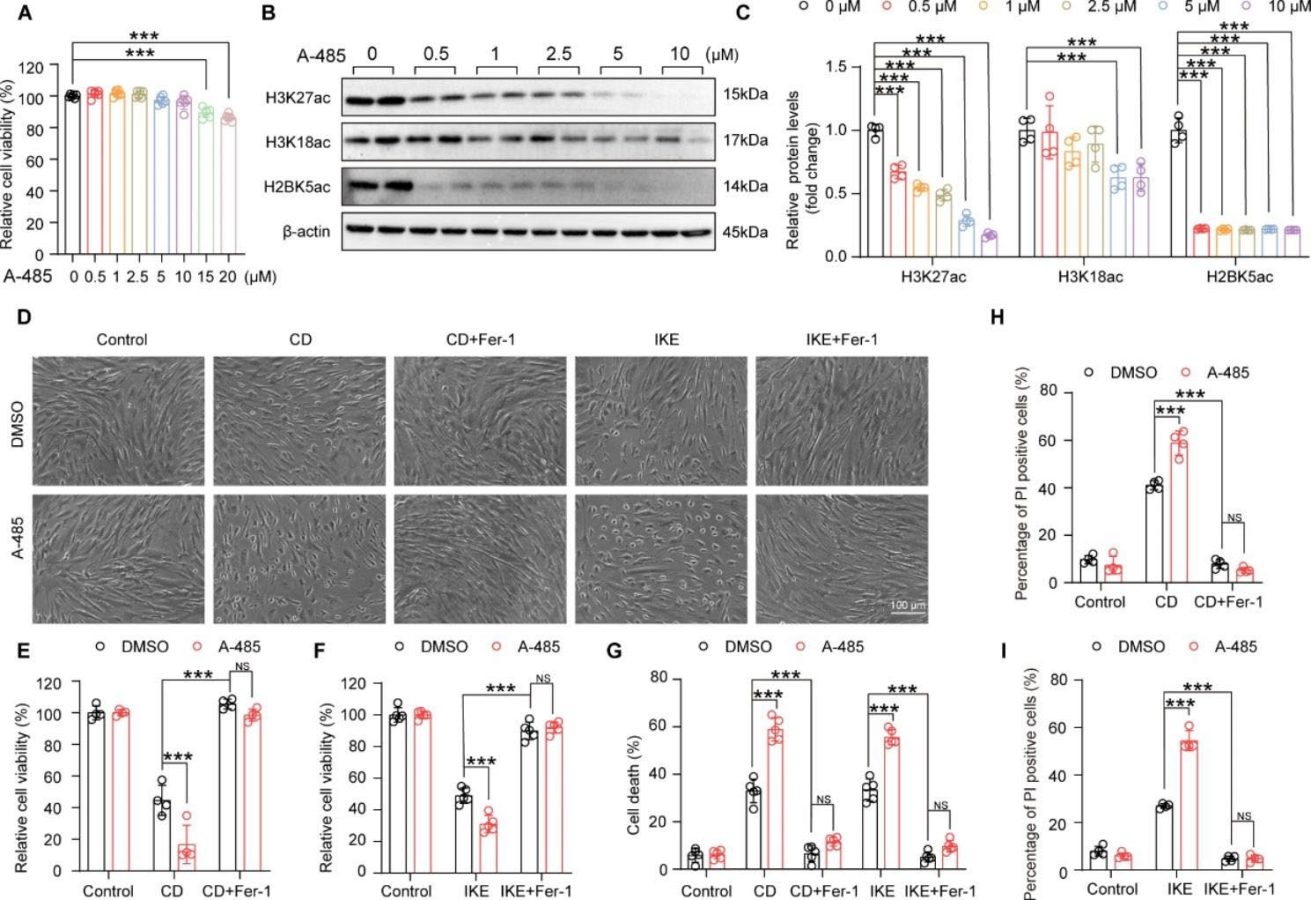
A-485 reduced cell viability and increased cell death of HASMCs treated with CD and IKE. **A.** A CCK8 assay was performed to show the relative cell viability detected in HASMCs after treatment with different concentrations of A-485 (0, 0.5, 1, 2.5, 5, 10, 15 and 20 µM) (n = 6 per group). **B-C.** H3K27ac, H3K18ac, and H2BK5ac protein levels in HASMCs treated with A-485 at different concentrations of 0, 0.5, 1, 2.5, 5 and 10 µM were measured by western blot (n = 4 per group). β-actin served as a loading control. **D.** Representative images showing cell death after treatment with DMSO and A-485 under the ferroptosis models induced by CD and IKE, alone or in combination with Fer-1. **E-F.** The CCK8 assay showed the relative viability of HASMCs treated with DMSO and A-485 after CD (E), IKE (F), alone or in combination with Fer-1 stimulation for the indicated time (E: n = 4 per group, F: n = 5 per group). **G.** An LDH assay was performed to examine the cell death of HASMCs after treating as described above (n = 5 per group). **H-I.** Flow cytometry with PI staining showed the percentage of PI positive cells after treatment with DMSO and A-485 under the ferroptosis models induced by CD (H), IKE (I), alone or in combination with Fer-1 (n = 4 per group). Values are means ± SD; ***p < 0.001, NS, no significant

Next, we were curious about whether the acetyltransferase activity of P300 is indispensable for its function in ferroptosis. A-485 is a small molecule bound to the catalytic active site of P300 and inhibits the acetyltransferase activity of P300 by competing with acetyl coenzyme A (Lasko et al. 2017). To investigate the effects of A-485 on HASMCs, the optimal drug concentration was first evaluated by culturing the cells with different concentrations of A-485 (0, 0.5, 1, 2.5, 5, 10, 15, and 20 µM) and then assessing the cell cytotoxicity using a CCK-8 assay. The results showed that concentration over 15 µM A-485 displayed distinct cytotoxicity after treatment for 24 h **(**Fig. 4A**)**. Then, the acetylation levels of H3K27, H3K18, and H2BK5, which were reported to be substrates of P300, were detected in HASMCs treated with 0, 0.5, 2.5, 5 or 10 µM A-485 to evaluate its inhibitory effects. Although the acetylation levels of H3K27 and H2BK5 were strikingly reduced at concentrations as low as 0.5 µM for A-485, H3K18ac could be inhibited only at concentrations greater than 5 µM **(**Fig. 4B-C**)**. Thus, given that 5 µM A-485 treatment had a satisfactory inhibitory effect without cytotoxicity, 5 µM A-485 was used for subsequent experiments in HASMCs.

**Fig. 5:**
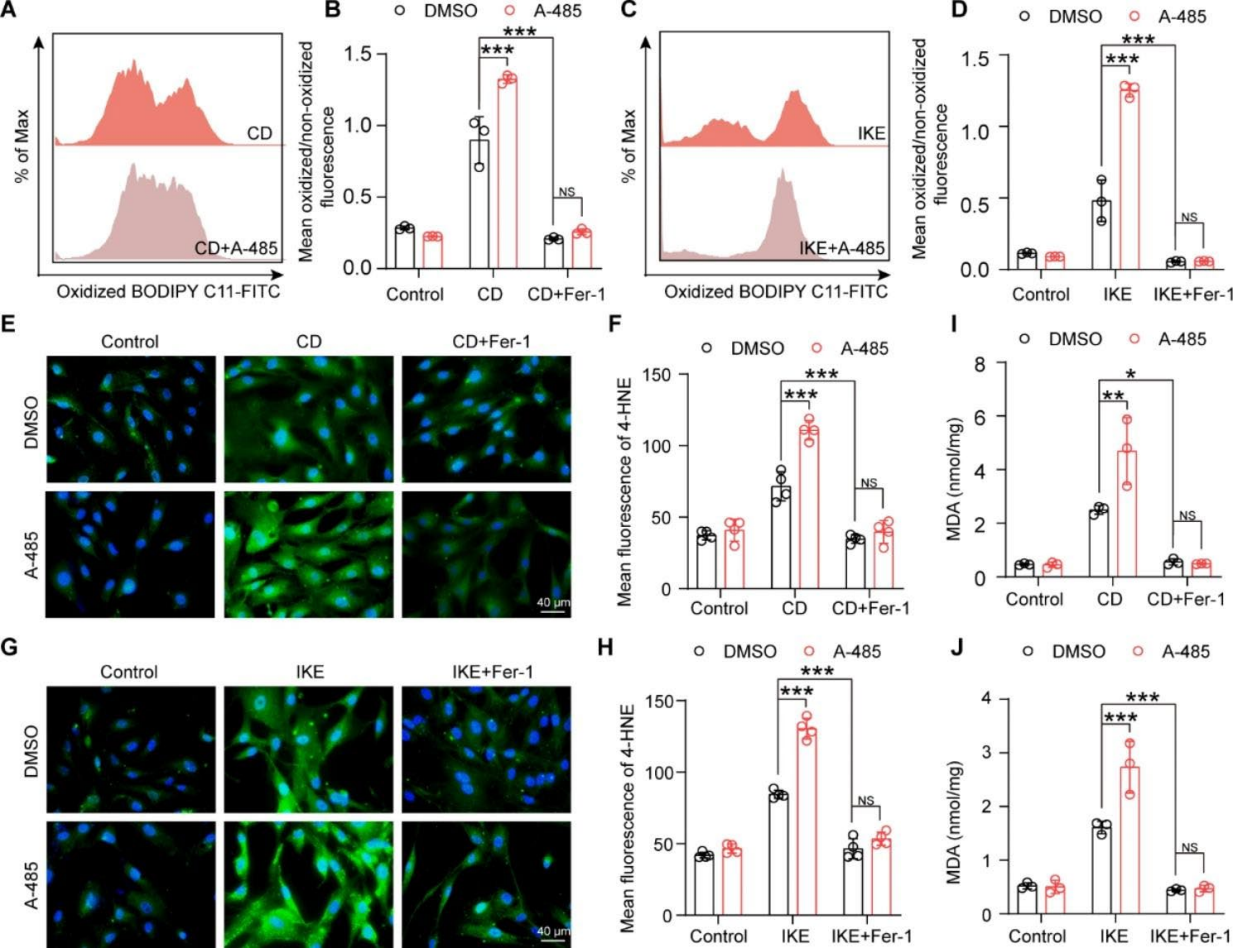
A-485 aggravated the accumulation of lipid peroxidation during HASMC ferroptosis. **A-D.** The ratio of oxidized BODIPY-C11/non-oxidized BODIPY-C11 fluorescence detected by the BODIPY-C11 kit after treatment with DMSO and A-485 under the ferroptosis models induced by CD (A-B), IKE (C-D), alone or in combination with Fer-1 (n = 3 per group). **E and G.** Representative images of 4-HNE immunofluorescence staining in HASMCs treated with DMSO and A-485 after CD (E), IKE (G), alone or in combination with Fer-1 stimulation for the indicated time. **F and H.** Quantitative analysis of 4-HNE immunofluorescence staining (n = 4 per group). **I-J.** The level of MDA in HASMCs treated as described above was examined by an MDA assay kit (n = 3 per group). Values are means ± SD; ***p < 0.001, **p < 0.01, *p < 0.05, NS, no significant

We next evaluated the effects of A-485 in ferroptosis of HASMCs. As shown in the cell images, compared with CD or IKE treatment alone, the number of dead cells significantly increased after HASMCs were challenged with A-485 and CD/IKE **(**Fig. 4D**)**. Consistent with this result, the results of the CCK-8 assay, LDH release assay and PI staining demonstrated that A-485 treatment decreased cell viability and increased cell injury and death induced by CD and IKE **(**Fig. 4E- I**)**. Furthermore, the results of the BODIPY-C11 assay, 4-HNE staining, and MDA assay showed that CD- and IKE-induced lipid peroxidation was also exacerbated by A-485 stimulation in HASMCs **(**Fig. 5A-J**)**. Similarly, Fer-1 largely eliminated the effect of CD, IKE and their synergistic aggravated effects with A-485 on cell viability and lipid peroxidation in HASMCs. Therefore, the results of A-485 suggested that the acetyltransferase activity of P300 is necessary for its role in ferroptosis of VSMCs.

### P300 and P53 competitively bind HIF-1α to regulate HMOX1 expression during ferroptosis of HASMCs

**Fig. 6:**
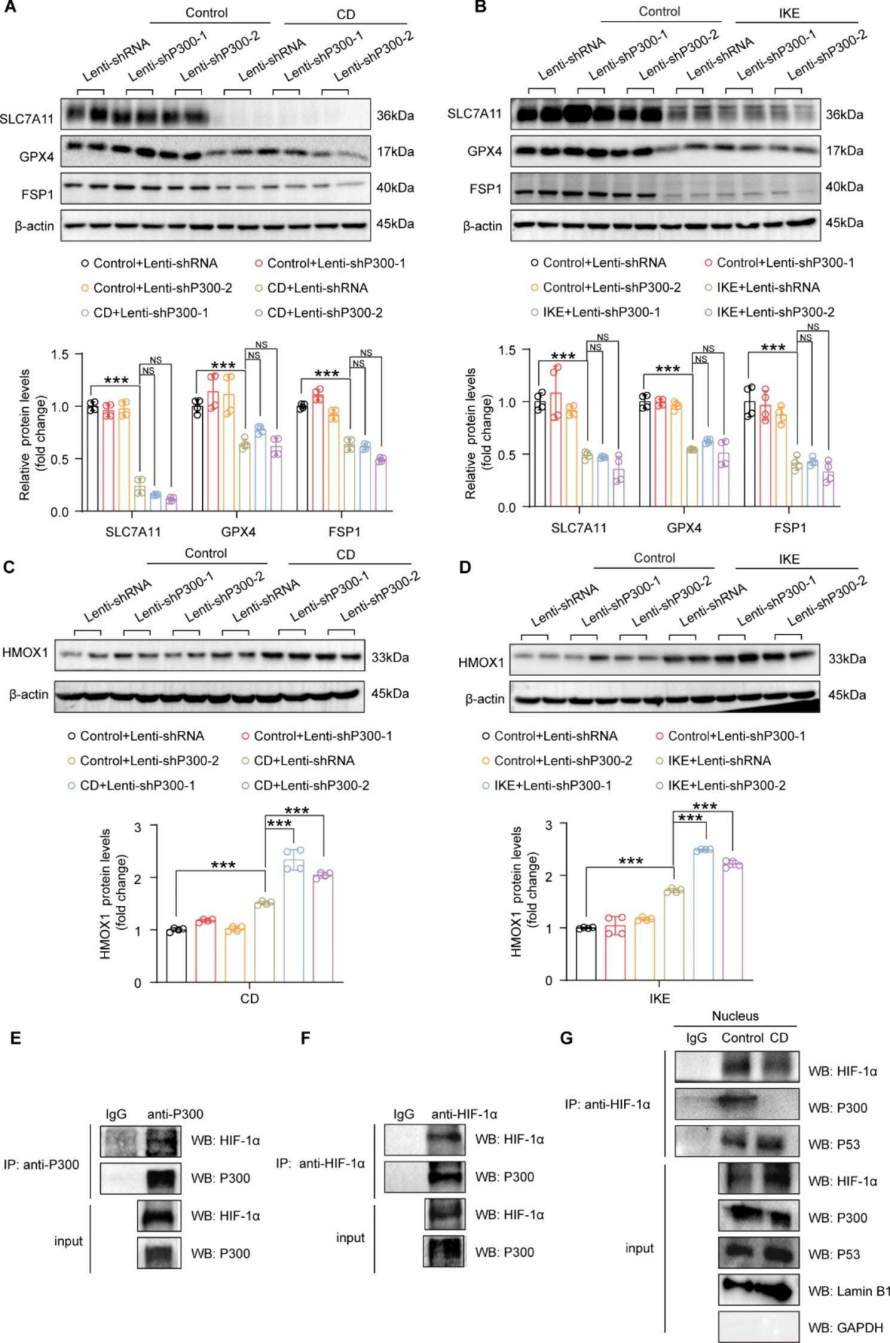
P300 and P53 competitively bind HIF-1α to regulate HMOX1 expression during ferroptosis of HASMCs. **A-B.** Western blot analysis and quantification results showing SLC7A11, GPX4, and FSP1 protein levels after CD (A), and IKE (B) stimulation in HASMCs infected with lenti-shRNA, lenti-shP300-1 and lenti-shP300-2 (A-B), β-actin served as a loading control (n = 4 per group). **C-D**. Western blot analysis and quantification results showing HMOX1 protein levels in HASMCs infected with lenti-shRNA, lenti-shP300-1 and lenti-shP300-2 after CD and IKE stimulation. β-actin served as a loading control (n = 4 per group). **E**. Co-immunoprecipitation results showed that endogenous P300 interacted with exogeneous HIF-1α in HASMCs after treatment with MG132. **F**. The interaction between endogenous HIF-1α and exogenous P300 in 293T cells after treatment with MG132 was verified by Co-immunoprecipitation. **G**. Co-immunoprecipitation showed the interaction between endogenous HIF-1α and endogenous P300 and P53 after treatment with CD and MG132 in nucleus of HASMCs. Values are means ± SD; ***p < 0.001, NS, no significant

To further investigate the molecular mechanisms that mediate the function of P300 in ferroptosis of HASMCs, we first evaluated the protein levels of SLC7A11, FSP1 and GPX4, which are the key regulators of ferroptosis, in HASMCs with P300 knockdown under CD and IKE treatment. The results showed that compared with the control, the protein levels of SLC7A11, FSP1 and GPX4 significantly decreased after treatment with CD and IKE **(**Fig. 6A and B**)**. Unexpectedly, comparable protein levels of SLC7A11, FSP1 and GPX4 were found between HASMCs with or without P300 knockdown under CD and IKE treatment **(**Fig. 6A and B**)**, which indicated that SLC7A11, FSP1 and GPX4 might not mediate the regulatory effects of P300 in ferroptosis of HASMCs. As HMOX1 catalyzes the degradation of heme and releases ferrous iron to aggravate ferroptosis (Gamage et al. 2021), we further analyzed whether P300 affects HMOX1 expression. Our results demonstrated that both CD and IKE treatment noticeably upregulated HMOX1 protein levels, and P300 knockdown further enhanced its expression **(**Fig. 6C and D**)**. It has been reported that HIF-1α functions as a transcription factor and directly regulates HMOX1 expression during ferroptosis (Wu et al. 2022b). Thus, we were curious about whether P300 interacts with HIF-1α to regulate HMOX1. The results of co-IP showed that P300 bound to HIF-1α and vice versa under normal conditions **(**Fig. 6E-F**)**. On the other hand, the transcriptional activity of HIF-1α is also affected by P53 (Parandavar and Yazdanparast 2017). We further analyzed the interaction among HIF-1α, P300, and P53 in nuclear protein extraction of HASMCs treated with or without CD. The results of endogenous co-IP indicated that HIF-1α interacted with both P300 and P53 under normal conditions, while the interaction between HIF-1α and P300 was decreased and the interaction between HIF-1α and P53 was enhanced after CD treatment in the nucleus **(**Fig. 6G**)**. These results indicated that P300 and P53 competitively combined with HIF-1α to regulate the expression of HMOX1 affecting ferroptosis of HASMCs.

### HIF-1α inhibition abolished the pro-ferroptotic effects of P300 deficiency in HASMCs

**Fig. 7:**
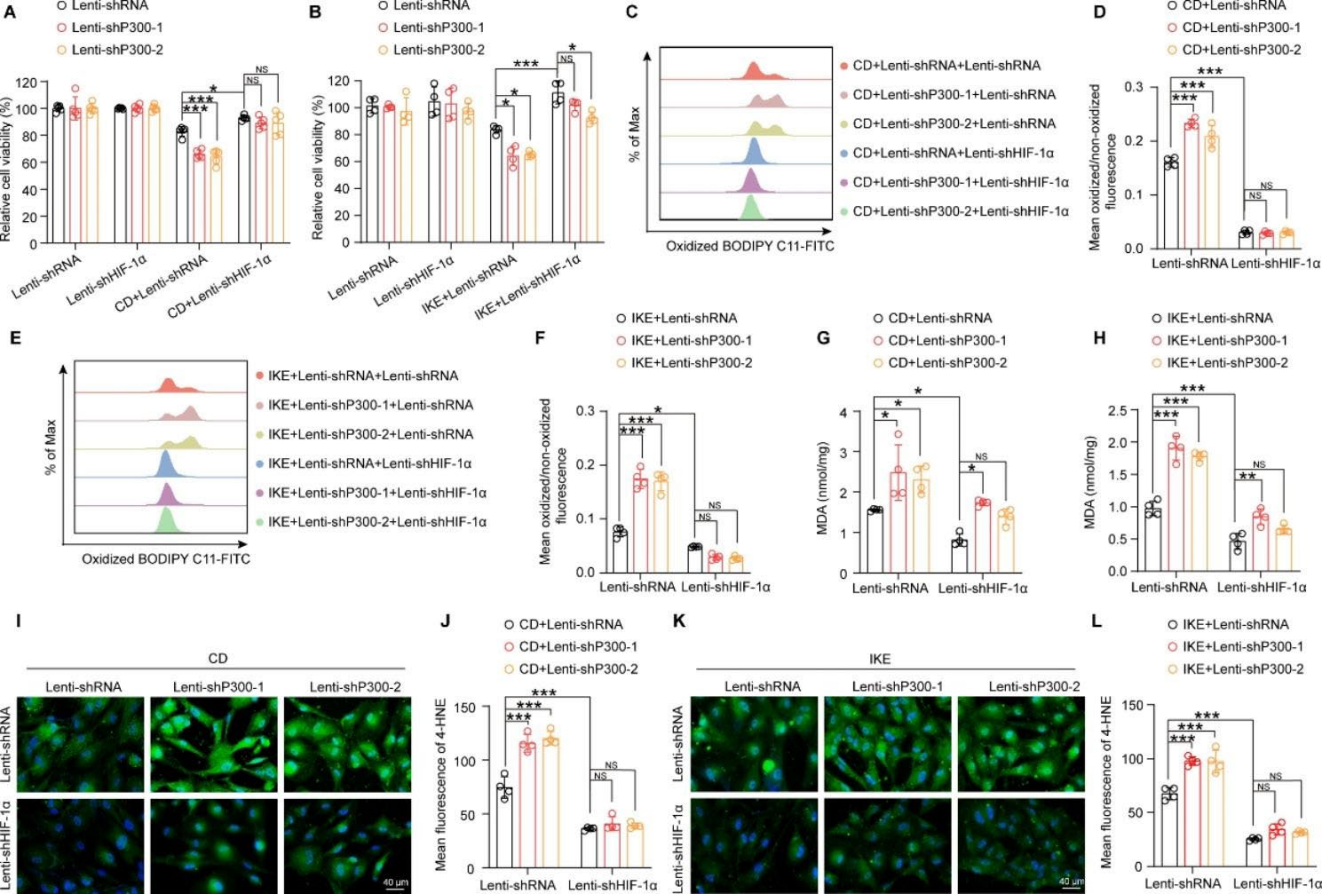
HIF-1α downregulation abolished the pro-ferroptotic effects of P300 deficiency in HASMCs. P300 knockdown HASMCs were infected with lenti-shRNA and lenti-shHIF-1α, and then these HASMCs were used for subsequent experiments. **A-B.** Relative cell viability was evaluated by CCK8 kit in HASMCs after treatment with CD (A) and IKE (B) (n = 5 per group). **C-F.** The level of lipid ROS (oxidized BODIPY-C11/non-oxidized BODIPY-C11 fluorescence ratio) examined by the BODIPY-C11 kit in HASMCs treated with CD (C-D) and IKE (E-F) (n = 4 per group). **G-H.** An MDA assay was used to detect the MDA levels of HASMCs after treatment with CD (G) and IKE (H) (n = 4 per group). **I-L.** Representative images of immunofluorescence staining and quantitative analysis of 4-HNE in HASMCs among the indicated groups (n = 4 per group). Values are means ± SD; ***p < 0.001, **p < 0.01, *p < 0.05, NS, no significant

To further verify the hypothesis that HIF-1α mediates the effects of P300 on VSMC ferroptosis, HIF-1α was knocked down to reverse the function of P300. The results showed that HIF-1α knockdown significantly improved the viability of cell injured by CD and IKE treatment **(**Fig. 7A and B**)**. Lipid peroxidation evaluation showed that HIF-1α deficiency inhibited CD- and IKE-induced lipid peroxidation, as evidenced by the reduced ratio of oxidized to non-oxidized lipids **(**Fig. 7C-F**)** and the levels of MDA **(**Fig. 7G-H**)** and 4-HNE **(**Fig. 7I-L**)**. More importantly, knockdown of HIF-1α largely reversed the pro-ferroptotic effects of P300 in HASMCs **(**Fig. 7A-L**)**.

**Fig. 8:**
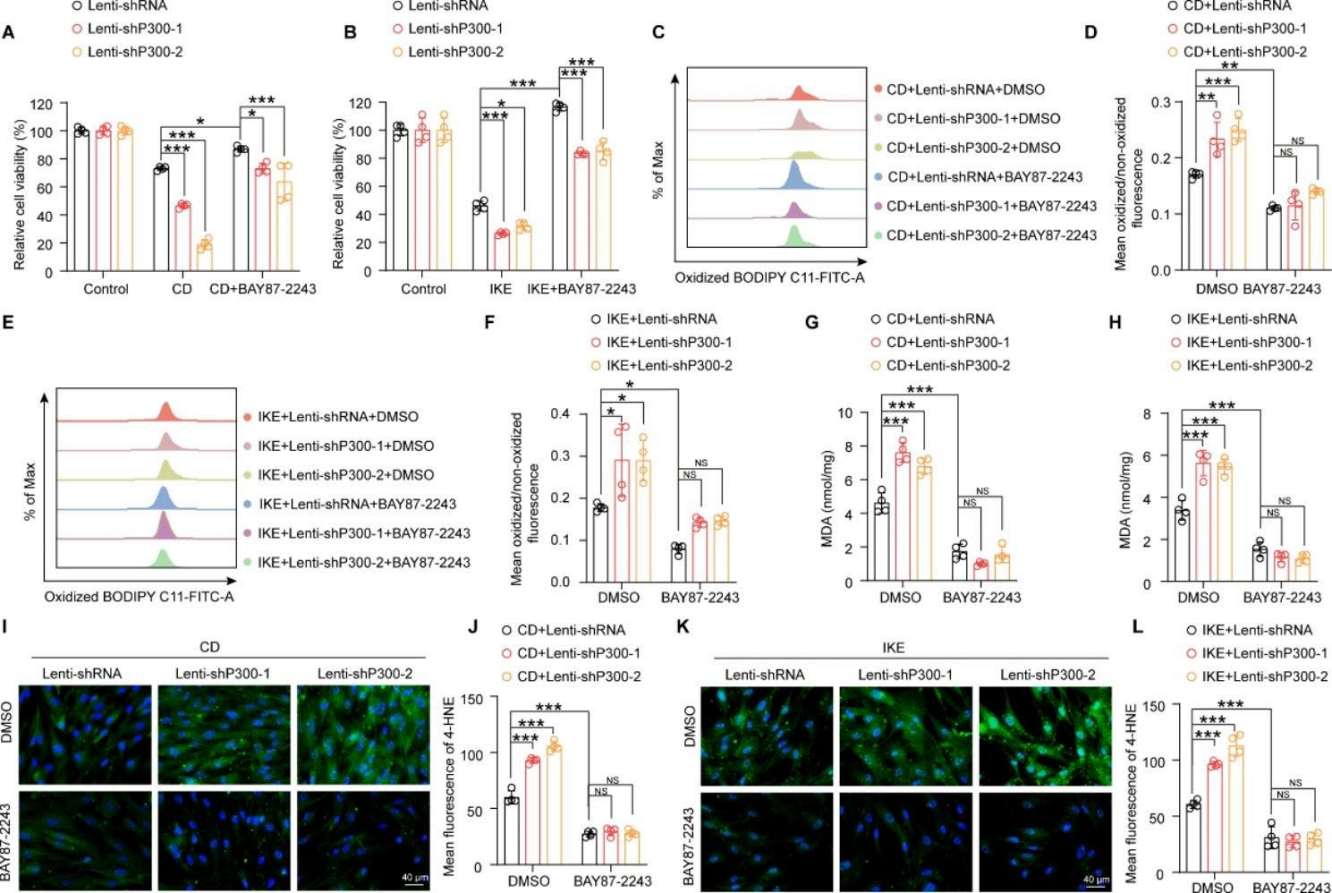
HIF-1α inhibition eliminated the pro-ferroptotic effects of P300 deficiency in HASMCs. **A-B.** Relative cell viability of HASMCs infected with lenti-shRNA, lenti-shP300-1, and lenti-shP300-2 by using a CCK8 kit after treatment with CD (A) and IKE (B) in the presence or absence of BAY87-2243 (n = 4 per group). **C-F.** The oxidized BODIPY-C11/non-oxidized BODIPY-C11 fluorescence ratio was evaluated by BODIPY-C11 kit in the indicated lentivirus-infected HASMCs treated with DMSO and BAY87-2243 after CD (C-D) and IKE (E-F) stimulation for 16 h (n = 4 per group). **G-H.** An MDA assay was used to assess MDA levels in indicated lentivirus-infected HASMCs treated with DMSO and BAY87-2243 after CD (G) and IKE (H) stimulation for the indicated time (n = 4 per group). **I-L.** Representative images of immunofluorescence staining and quantitative analysis of 4-HNE in HASMCs among the indicated groups in the presence or absence of BAY87-2243 (n = 4 per group). Values are means ± SD; ***p < 0.001, **p < 0.01, *p < 0.05, NS, no significant

Given that the activity of HIF-1α is critical for its function, BAY87-2243, an inhibitor of HIF-1α that suppresses hypoxia-induced gene activation (Ellinghaus et al. 2013), was used to validate the need for HIF-1α activity for its role in ferroptosis. Consistent with the results of HIF-1α knockdown, BAY87-2243 also inhibited CD- and IKE-induced ferroptosis, including improving cell viability and reducing lipid peroxidation **(**Fig. 8A-L**)**. Moreover, the detrimental effects of P300 knockdown on CD- and IKE-induced cell viability and lipid peroxidation were largely nullified by BAY87-2243 **(**Fig. 8A-L**)**.

Therefore, these results revealed that both HIF-1α knockdown and inhibition can offset the pro-ferroptotic effects of P300 deficiency in HASMCs. The P300/HIF-1α/HMOX1 axis is a novel mechanism regulating ferroptosis.

## Discussion

**Fig. 9:**
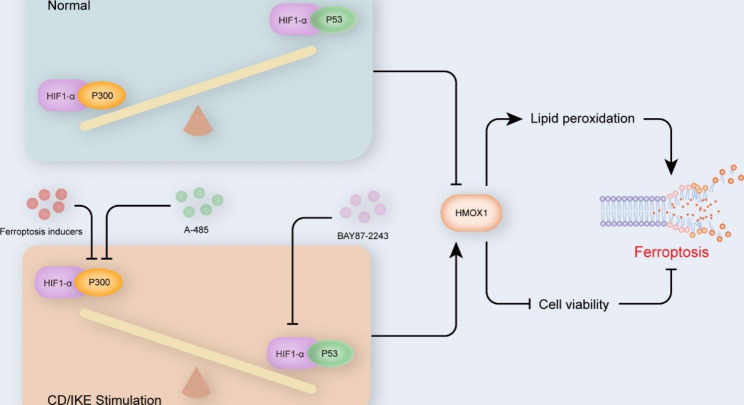
Schematic summary. P300 deficiency and inhibition induced VSMC ferroptosis by activating the HIF-1α/HMOX1 axis. P300 and P53 competitively bound HIF-1α to regulate HMOX1 expression. Under normal conditions, P300 interacted with HIF-1α to inhibit HMOX1 expression. When treatment with ferroptosis inducers, the decrease of P300 contributed to the interaction between P53 and HIF-1α to activate HMOX1 transcription. The activation of HMOX1 decreased cell viability and aggravated lipid peroxidation and further triggered ferroptosis

Recently, several breakthrough studies revealed that ferroptosis of VSMCs was a novel and critical pathological mechanism of aortic degeneration. However, the mechanisms mediating ferroptosis of VSMCs remain largely unclear. In this study, we revealed a novel function of P300 in regulating ferroptosis of VSMCs **(**Fig. 9**)**. The expression level of P300 was significantly decreased in HASMCs treated with CD and IKE. Knockdown of P300 or inhibition of P300 activity by A-485 aggravated ferroptosis of VSMCs. Furthermore, we found that the HIF-1α/HMOX1 axis, but not a classic ferroptosis pathway, is responsible for the impacts of P300 on ferroptosis of VSMCs.

In the present study, we revealed that acetyltransferase P300 deficiency promoted ferroptosis of VSMCs, which indicated that P300 may play critical roles in the vascular diseases related to VSMC ferroptosis. In the recent years, increasing evidences revealed that ferroptosis of VSMCs was closely associated with a variety of vascular diseases. For example, our latest studies demonstrated that VSMC ferroptosis is an important factor resulting in VSMC loss during the development of aortic dissection (AD), and inhibition of ferroptosis with the classical inhibitor (liproxstatin-1) or novel inhibitor (BRD4770) of ferroptosis are largely eliminated the β-aminopropionitrile-induced development and rupture of AD in mice (Chen et al. 2022b; Li et al. 2022). Moreover, cigarette smoking is a well-known risk factor for cardiovascular diseases, cigarette smoke extract was reported to induce VSMC ferroptosis (Sampilvanjil et al. 2020). These evidences revealed that VSMC ferroptosis is a novel and critical pathological mechanism involved in aortic aneurysm and AD development. Vascular calcification and atherosclerosis are risk factors for many cardiovascular diseases, including aortic aneurysm and AD. Inhibition of ferroptosis by ferrostatin-1 remarkably diminished calcification of rat and human arterial rings in vitro and also significantly attenuated aortic calcification in vitamin D3-overloaded mice in vivo (Ye et al. 2022). In the study of ferroptosis-related differentially expressed genes of atherosclerosis, HMOX1 was found to be remarkably increased and its inhibitor protected HASMCs from erastin-induced ferroptosis (Wu et al. 2022a). Additionally, Zhang et al. demonstrated that RAS-selective lethal 3 (RSL3), a ferroptosis activator, promoted VSMC phenotypic switching and aggravated neointimal hyperplasia in mice (Zhang et al. 2022). Therefore, P300 regulated VSMC ferroptosis may participate in the development of degenerative vascular disease, and activation of P300 is expected to prevent and treat these related diseases. However, more in-depth in vivo studies are needed to validate this hypothesis.

As reported previously, the regulation of ferroptosis is mostly related to redox systems, and inactivation of the lipid peroxide repair network including the axes of NADPH-FSP1 (ferroptosis suppressor protein 1)-CoQ10 (coenzyme Q10), GCH1 (GTP cyclohydrolase 1)-BH4 (tetrahydrobiopterin) and glutathione-GPX4, which are the classic metabolic pathways of ferroptosis (Wei et al. 2020). In the present study, comparable protein levels of SLC7A11, FSP1 and GPX4 were found between HASMCs with or without P300 knockdown, which indicated that SLC7A11, FSP1 and GPX4 might not mediate the regulatory effects of P300 on ferroptosis of HASMCs. Interestingly, we found that under normal conditions, P300, HIF-1α and P53 form a complex, but ferroptosis inducer CD treatment diminishes the interaction of P300 with HIF-1α and enhances the interaction of HIF-1α and P53. It is reported that P300 banded with HIF-1α to regulate the transcription of the hypoxia-responsive genes (e.g., vascular endothelial growth factor and the glycolytic enzyme lactate dehydrogenase) (Zakrzewska et al. 2005). As we known, HIF-1α is unstable and rapidly degraded via the von Hip-pel-Lindau tumor suppressor gene product-mediated ubiquitin-proteasome pathway under normoxia (Lee et al. 2004). P53 serve as a molecular chaperone for HIF-1α to stabilize HIF-1α and then promotes its binding to DNA response elements (Madan et al. 2019). However, the roles of HIF-1α and P53 in ferroptosis is controversial. For example, HIF-1α could promote ferroptosis by regulating SLC7A11 in hepatic stellate cell or HMOX1 in Leydig and Sertoli cell of testes, and it also was reported to inhibit ferroptosis by activating the Hippo‑YAP signaling pathway in non‑small cell lung cancer or lncRNA-PMAN in gastric cancer (Lin et al. 2022; Wu et al. 2022b; Yuan et al. 2022; Zheng et al. 2023). Similarly, P53 could enhance cellular susceptibility to ferroptosis by regulating SLC7A11 in human bronchial epithelial cells, but delays the ferroptosis by banding to DPP4 in cancer cells (Xie et al. 2017; Yang et al. 2022). In the present study, we demonstrated that P300 competes with P53 to bind HIF-1α and inhibits the transcriptional activity of HIF-1α on HMOX1 to alleviate ferroptosis of VSMCs, and HIF-1α knockdown or inhibition largely reversed the effects of P300 deficiency on ferroptosis. Thus, despite the heterogeneity of HIF-1α and P53 functions in different cell types, we revealed that their interaction upregulates the expression of HMOX1 to promote ferroptosis, at least in VSMCs.

Both CD and IKE treatment noticeably upregulated HMOX1 protein levels, which was further enhanced by P300 knockdown in VSMCs. HMOX1, a dual regulator of iron and ROS homeostasis, was suggested to play an important role in ferroptosis. The activation of HMOX1 triggered ferroptosis through iron overloading and subsequently excessive ROS generation and lipid peroxidation (Chiang et al. 2021). The pro-ferroptotic role of HMOX1 has been validated by several studies. For example, Hassannia et al. reported that HMOX1-mediated increases in the labile Fe (II) pool boosted ferroptosis(Hassannia et al. 2018). ZnPP, an inhibitor of HMOX1, protected HASMCs from Erastin-induced ferroptosis (Wu et al. 2022a). We further revealed that the pro-ferroptotic role of P300 deficiency in VSMCs was mediated by HIF-1α/HMOX1 axis. Feng et al. reported that HIF-1α and HMOX1 levels significantly increased during ferroptosis induced by kidneys diabetic nephropathy, and proposed that ferroptosis might enhance diabetic nephropathy through HIF-1α/HMOX1 pathway (Feng et al. 2021). HIF-1α knockout rescued Di- (2-ethylhexyl) phthalate-induced ferroptosis via regulating HMOX1 transcription by binding to the HMOX1 promoter region (Wu et al. 2022b). These results suggest that HIF-1α/HMOX1 axis is a critical mechanism affecting ferroptosis by regulating iron metabolism. Furthermore, activation of inflammatory signaling pathways also promotes the expression of HMOX1, which in turn induces ferroptosis (Chen et al. 2023; El-Shitany and Eid 2019). P300, as an important coactivator, was reported to control transcriptional activity and expression of TNF-α (Granja et al. 2006; Yang et al. 2014). TNF-α is a cytokine activating inflammatory signaling pathways, including NF-κB pathway, which have been reported to involve in the regulation of ferroptosis (Jankauskas et al. 2023; Pooladanda et al. 2019). However, whether P300 affects ferroptosis by regulating TNF-α expression and inflammatory signaling pathways needs further investigation.

In the present study, although we have elucidated the effect of P300 on ferroptosis through numerous in vitro experiments, several issues remain to be investigated in depth. First, more in vivo experiments are needed to verify the involvement of P300-regulated VMSC ferroptosis in the development of cardiovascular diseases, such as vascular calcification, atherosclerosis, aortic aneurysm and AD. Second, we found that the expression of P300 was significantly reduced in VSMCs treated with ferroptosis inducers, but the upstream mechanisms regulating the reduction of P300 remains unclear.

## Conclusions

In conclusion, we revealed a novel role of P300 in CD- and IKE-induced VSMC ferroptosis, that is, P300 knockdown or inhibition by A-485 accelerates VSMC ferroptosis by activating the HIF-1α/HMOX1 axis. These findings provide new insights into the mechanisms of ferroptosis and may have significant implications for the development of novel strategies to treat the diseases related to VSMC ferroptosis (e.g., aortic dissection) by modulating P300.

## Electronic supplementary material

Below is the link to the electronic supplementary material.

Supplementary Material 1

## Data Availability

All data generated or analyzed during this study are included in this published article.

## List of abbreviations

ACTA2: Smooth muscle actin
BH4: Tetrahydrobiopterin
CBP: CREB binding protein
CCK-8: Cell Counting Kit-8
CD: Cystine deprivation
CNN1: Calponin
Co-IP: Co-Immunoprecipitation
CoQ10: Coenzyme Q10
Fer-1: Ferrostatin-1
FSP1: Ferroptosis suppressor protein 1
GCH1: GTP cyclohydrolase 1
GPX4: Glutathione peroxidase 4
H2BK5: Histone H2B lysine 5
H3K18: Histone H3 lysine 18
H3K18ac: Acetylated histone H3 lysine 18
H3K23ac: Acetylated histone H3 lysine 23
H3K27: Histone H3 lysine 27
H4K12ac: Acetylated histone H4 lysine 12
H4K8ac: Acetylated histone H4 lysine 8
H4K5ac: Acetylated histone H4 lysine 5
HASMCs: Human aortic smooth muscle cells
Herpud1: Homocysteine-responsive endoplasmic reticulum-resident ubiquitin-like domain member 1
HIF-1α: Hypoxia-inducible factor-1α
HMOX1: Heme oxygenase 1
4-HNE: 4-hydroxynonenal
IKE: Imidazole ketone erastin
LDH: Lactate dehydrogenase
3-MA: 3-Methyladenine
MDA: Malondialdehyde assay
MYH11: Smooth muscle myosin heavy chain
NADPH: Nicotinamide adenine dinucleotide phosphate OD:Optical density
P300: E1A-associated 300-kDa protein
PI: Propidium iodide
ROS: Reactive oxygen species
ShRNA: Short hairpin RNA
SLC7A11: Solute carrier family 7 member 11
TAAD: Stanford type A aortic dissection
TBA: Thiobarbituric acid
TBST: Tris buffered saline tween
TFR: Transferrin receptor
VSMCs: Vascular smooth muscle cells
XBP1s: X-box binding protein 1
