# Supplementary material for: Histone acetyltransferase P300 deficiency promotes ferroptosis of vascular smooth muscle cells by activating the HIF-1α/HMOX1 axis
Source: Mol Med. 2023 Jul 6;29:91. doi: 10.1186/s10020-023-00694-7 (PMC10324182; doi:10.1186/s10020-023-00694-7)
Supplement: Supplementary file 1 — Supplementary Material 1 [file 10020_2023_694_MOESM1_ESM.docx]

**Response to Reviewers’ Comments**

**Manuscript ID:** MOME-D-23-00245

**Manuscript TITLE:** Histone Acetyltransferase P300 Deficiency Promotes Ferroptosis of Vascular Smooth Muscle Cells by Activating the HIF-1α/HMOX1 Axis

We greatly thank the reviewers and editors for their hard work and their critical and insightful comments on our manuscript. Overall, we appreciated the constructive comments made by the reviewers, which have helped us to improve our manuscript. Accordingly, the manuscript has been revised, and all of the revised sections are highlighted in red. We hope that the reviewers will find that our revised manuscript is improved and acceptable in *Molecular Medicine.*

**Reviewer 1#:**

**Comments**

**Q1).	The abstract does not give the immediate picture of how the study was conducted and its main results.**

**Re:** Thank you for this valuable comment. We apologized for such an indistinct abstract and have re-edited the abstract in our revised manuscript.

**Abstract**

**Background****:** E1A-associated 300-kDa protein (P300), an endogenous histone acetyltransferase, contributes to modifications of the chromatin landscape of genes involved in multiple cardiovascular diseases. Ferroptosis of vascular smooth muscle cells (VSMCs) is a novel pathological mechanism of aortic dissection. However, whether P300 regulates VSMC ferroptosis remains unknown.

**Methods:** Cystine deprivation (CD) and imidazole ketone erastin (IKE) were used to induce VSMC ferroptosis. Two different knockdown plasmids targeting P300 and A-485 (a specific inhibitor of P300) were used to investigate the function of P300 in the ferroptosis of human aortic smooth muscle cells (HASMCs). Cell counting kit-8, lactate dehydrogenase and flow cytometry with propidium iodide staining were performed to assess the cell viability and death under the treatment of CD and IKE. BODIPY-C11 assay, immunofluorescence staining of 4-hydroxynonenal and malondialdehyde assay were conducted to detect the level of lipid peroxidation. Furthermore, co-immunoprecipitation was utilized to explore the interaction between P300 and HIF-1α, HIF-1α and P53.

**Results:** Compared with normal control, the protein level of P300 was significantly decreased in HASMCs treated with CD and IKE, which was largely nullified by the ferroptosis inhibitor ferrostatin-1 but not by the autophagy inhibitor or apoptosis inhibitor. Knockdown of P300 by short-hairpin RNA or inhibition of P300 activity by A-485 promoted CD- and IKE-induced HASMC ferroptosis, as evidenced by a reduction in cell viability and aggravation of lipid peroxidation in HASMCs. Furthermore, we found that hypoxia-inducible factor-1α (HIF-1α)/heme oxygenase 1 (HMOX1) pathway was responsible for the impacts of P300 on ferroptosis of HASMCs. The results of co-immunoprecipitation demonstrated that P300 and P53 competitively bound HIF-1α to regulate the expression of HMOX1. Under normal conditions, P300 interacted with HIF-1α to inhibit HMOX1 expression, while reduced expression of P300 induced by ferroptosis inducers would favor HIF-1α binding to P53 to trigger HMOX1 overexpression. Furthermore, the aggravated effects of P300 knockdown on HASMC ferroptosis were largely nullified by HIF-1α knockdown or the HIF-1α inhibitor BAY87-2243.

**Conclusion:** Thus, our results revealed that P300 deficiency or inactivation facilitated CD- and IKE-induced VSMC ferroptosis by activating the HIF-1α/HMOX1 axis, which may contribute to the development of diseases related to VSMC ferroptosis.

**Q2). Given the importance of TNF-alpha in the pathophysiology of ferroptosis (doi:** **10.3390/antiox12020326) and the know relationship linking TNF-alpha and P300 (10.1152/ajplung.00320.2013; doi:** **10.4049/jimmunol.176.1.451)**

**The following pertinent reports should be mentioned/discussed:**

**doi: 10.1152/ajplung.00320.2013**

**doi: 10.4049/jimmunol.176.1.451**

**doi: 10.3390/antiox12020326.**

**doi: 10.3389/fcvm.2022.833642.**

**doi: 10.1186/s10020-022-00549-7.**

**doi: 10.3390/cells10092401.**

**doi: 10.3389/fendo.2021.626390.**

**Re:** Thanks for these insightful suggestions. As this reviewer said that TNF-α is closely related to ferroptosis, and P300 is one of the regulators that controls the expression of TNF-α. TNF-α is an inflammatory factor that activates multiple inflammatory signaling pathways, including the NF-κB pathway, and our recent study confirmed that activation of inflammatory signaling pathways, such as NF-κB, promotes ferroptosis (Chen et al., 2022; Pooladanda et al., 2019). On the other hand, the activation of inflammatory signaling pathways also promotes the expression of HMOX1 and leads to the disorder of iron metabolism, which in turn induces ferroptosis (El-Shitany and Eid, 2019). Therefore, it is possible that P300 also affects ferroptosis by regulating the expression of TNF-α. As suggested by the reviewer, we have added detailed discussion about this issue and cited these references in the revised manuscript **(lines 2-5, lines 8-14, and lines 17-22, Page 27; lines 1-3, Page 28).**

“Both CD and IKE treatment noticeably upregulated HMOX1 protein levels, which was further enhanced by P300 knockdown in VSMCs. HMOX1, a dual regulator of iron and ROS homeostasis, was suggested to play an important role in ferroptosis. The activation of HMOX1 triggered ferroptosis through iron overloading and subsequently excessive ROS generation and lipid peroxidation (Chiang et al., 2021). The pro-ferroptotic role of HMOX1 has been validated by several studies. For example, Hassannia et al. reported that HMOX1-mediated increases in the labile Fe (II) pool boosted ferroptosis (Hassannia et al., 2018). ZnPP, an inhibitor of HMOX1, protected HASMCs from Erastin-induced ferroptosis (Wu et al., 2022a). We further revealed that the pro-ferroptotic role of P300 deficiency in VSMCs was mediated by HIF-1α/HMOX1 axis. Feng et al. reported that HIF-1α and HMOX1 levels significantly increased during ferroptosis induced by kidneys diabetic nephropathy, and proposed that ferroptosis might enhance diabetic nephropathy through HIF-1α/HMOX1 pathway (Feng et al., 2021). HIF-1α knockout rescued Di- (2-ethylhexyl) phthalate-induced ferroptosis via regulating HMOX1 transcription by binding to the HMOX1 promoter region (Wu et al., 2022b). These results suggest that HIF-1α/HMOX1 axis is a critical mechanism affecting ferroptosis by regulating iron metabolism. Furthermore, activation of inflammatory signaling pathways also promotes the expression of HMOX1, which in turn induces ferroptosis (Chen et al., 2023; El-Shitany and Eid, 2019). P300, as an important coactivator, was reported to control transcriptional activity and expression of TNF-α (Granja et al., 2006; Yang et al., 2014). TNF-α is a cytokine activating inflammatory signaling pathways, including NF-κB pathway, which have been reported to involve in the regulation of ferroptosis (Jankauskas et al., 2023; Pooladanda et al., 2019). However, whether P300 affects ferroptosis by regulating TNF-α expression and inflammatory signaling pathways needs further investigation.”

**References:**

1. Chiang, S.K., Chen, S.E., and Chang, L.C. The Role of HO-1 and Its Crosstalk with Oxidative Stress in Cancer Cell Survival. Cells. 2021; 10: 2401.
2. Hassannia, B., Wiernicki, B., Ingold, I., Qu, F., Van Herck, S., Tyurina, Y.Y., et al. Nano-targeted induction of dual ferroptotic mechanisms eradicates high-risk neuroblastoma. J Clin Invest. 2018; 128: 3341-3355.
3. Wu, D., Hu, Q., Wang, Y., Jin, M., Tao, Z., and Wan, J. Identification of HMOX1 as a Critical Ferroptosis-Related Gene in Atherosclerosis. Front Cardiovasc Med. 2022a; 9: 833642.
4. Feng, X., Wang, S., Sun, Z., Dong, H., Yu, H., Huang, M., et al. Ferroptosis Enhanced Diabetic Renal Tubular Injury via HIF-1alpha/HO-1 Pathway in db/db Mice. Front Endocrinol (Lausanne). 2021; 12: 626390.
5. Wu, Y., Wang, J., Zhao, T., Chen, J., Kang, L., Wei, Y., et al. Di-(2-ethylhexyl) phthalate exposure leads to ferroptosis via the HIF-1alpha/HO-1 signaling pathway in mouse testes. J Hazard Mater. 2022b; 426: 127807.
6. Chen, Y., Fang, Z.M., Yi, X., Wei, X., and Jiang, D.S. The interaction between ferroptosis and inflammatory signaling pathways. Cell Death Dis. 2023; 14: 205.
7. El-Shitany, N.A., and Eid, B.G. Icariin modulates carrageenan-induced acute inflammation through HO-1/Nrf2 and NF-kB signaling pathways. Biomed Pharmacother. 2019; 120: 109567.
8. Granja, A.G., Nogal, M.L., Hurtado, C., Del Aguila, C., Carrascosa, A.L., Salas, M.L., et al. The viral protein A238L inhibits TNF-alpha expression through a CBP/p300 transcriptional coactivators pathway. J Immunol. 2006; 176: 451-462.
9. Yang, C.M., Lee, I.T., Chi, P.L., Cheng, S.E., Hsiao, L.D., and Hsu, C.K. TNF-alpha induces cytosolic phospholipase A2 expression via Jak2/PDGFR-dependent Elk-1/p300 activation in human lung epithelial cells. Am J Physiol Lung Cell Mol Physiol. 2014; 306: L543-551.
10. Pooladanda, V., Thatikonda, S., Bale, S., Pattnaik, B., Sigalapalli, D.K., Bathini, N.B., et al. Nimbolide protects against endotoxin-induced acute respiratory distress syndrome by inhibiting TNF-alpha mediated NF-kappaB and HDAC-3 nuclear translocation. Cell Death Dis. 2019; 10: 81.
11. Jankauskas, S.S., Kansakar, U., Sardu, C., Varzideh, F., Avvisato, R., Wang, X., et al. COVID-19 Causes Ferroptosis and Oxidative Stress in Human Endothelial Cells. Antioxidants (Basel). 2023; 12: 326.
12. Zhang, S., Bei, Y., Huang, Y., Huang, Y., Hou, L., Zheng, X.L., et al. Induction of ferroptosis promotes vascular smooth muscle cell phenotypic switching and aggravates neointimal hyperplasia in mice. Mol Med. 2022; 28: 121.

**Q3). To ensure reproducibility by other laboratories, the manufacturer name and catalog number(s) of all the antibodies used should be clearly specified in the material and method section.**

**Re:** We apologized for missing some product information of reagents used in our present study. We have double checked the part of Methods and Materials, and have replenished all the missed information for all the reagents as suggested (Highlighted in red). Thank you for this insight suggestion.

**Q4).** **The paper is mainly descriptive and focused on its conclusions, not adequately acknowledging the limitations of the study. The strengths and limitations of the study should be deeply addressed, taking into account sources of potential bias or imprecision: Discuss both direction and magnitude of any potential bias.**

**Re:** We appreciated this valuable suggestion. We have discussed this in the revised version **(lines 4-10, Page 28 and lines 16-19, Page 28)**.

“In the present study, although we have elucidated the effect of P300 on ferroptosis through numerous in vitro experiments, several issues remain to be investigated in depth. First, more in vivo experiments are needed to verify the involvement of P300-regulated VMSC ferroptosis in the development of cardiovascular diseases, such as vascular calcification, atherosclerosis, aortic aneurysm and AD. Second, we found that the expression of P300 was significantly reduced in VSMCs treated with ferroptosis inducers, but the upstream mechanisms regulating the reduction of P300 remains unclear.

In conclusion, we revealed a novel role of P300 in CD- and IKE-induced VSMC ferroptosis, that is, P300 knockdown or inhibition by A-485 accelerates VSMC ferroptosis by activating the HIF-1α/HMOX1 axis. These findings provide new insights into the mechanisms of ferroptosis and may have significant implications for the development of novel strategies to treat the diseases related to VSMC ferroptosis (e.g., aortic dissection) by modulating P300.**”**

**Reviewer 2#:**

**Comments**

**Q1). In the figure 2A, why there are not included p300 protein levels in the nuclear fraction due to its nature as a transcriptional co-activator acting in the nucleus?**

**Re:** Thanks for the great suggestion. According to your suggestion, we extracted the proteins of nucleus and tested the knockdown efficiency of P300 in nucleus. The results showed that compared with lenti-shRNA control, both of shRNAs targeting P300 reduced the protein level of P300 in nucleus. The results were included in new **Figure 2B** in the revised manuscript **and show below**.

**Q2). It is interesting how p300 downregulation itself (Control) does not affect cell viability shown in Fig 2D, compared with those p300 inhibition data in Fig. 4A due to its implications on several cellular processes via interacting with a comprehensive range of proteins, how do you further discuss it.**

**Re:** We thank the reviewer for raising this concern. In Figure 4A, to screen the optimal working concentration of A-485, we treated the cells with different concentrations of A-485 (0, 0.5, 1, 2.5, 5, 10, 15, and 20 μM) for 24 h and then assessing the cell viability by using a CCK-8 assay. Meanwhile, we also detected the acetylation levels of P300 substrates by using western blot. The results of CCK-8 showed that concentrations of A-485 lower than 10 μM had little effect on cellular viability, while higher than 15 μM significantly reduced cellular viability **(Figure 4A and shown below)**. Moreover, the levels of P300 substrates, such as H3K18ac, H3K27ac and H2BK5ac, were largely reduced at concentrations higher than 5 μM of A-485 **(Figure 4B, 4C and shown below)**. Therefore, to reduce the cell death caused by drug toxicity as well as to increase the specificity of A-485 for targeting P300, we chose 5 μM as the final use concentration of A-485 in this study. As showed in **Figure 4D-4I**, 5 μM of A-485 treatment did not affect cell viability, which was consistent with the results of P300 knockdown in **Figure 2C-2H.**

There are two possible reasons why high concentrations of A-485 reduce cell viability: First, although A-485 is a specific inhibitor of P300, its specificity may decrease with increasing concentrations, leading to some unexpected off-target effects; Second, higher concentrations can also cause reduced cell viability due to cytotoxicity caused by drug overdose. Therefore, in order to ensure its specificity and reduce cytotoxicity, we chose a concentration of 5 μM that could significantly inhibit the acetylation levels of the substrates without toxicity to the cells for subsequent experiments.

**Figure 4:**

**Figure 2:**

**Q3). It is evident the interaction between HIF-1a and p53 in HASMCs treated with CD and MG132, however, it could be interesting to show also the immunoblot of p300 under these same conditions (endogenous p53 in Fig. 6G), to strengthen such mechanism of competence that is proposed. Why did not perform the** **co-immunoprecipitation using nuclear fraction?**

**Re:** We thank reviewer for raising this important issue. We have performed the co-immunoprecipitation using nuclear protein extraction. The results of endogenous co-IP indicated that HIF-1α interacted with both P300 and P53 under normal conditions, while the interaction between HIF-1α and P300 was decreased and the interaction between HIF-1α and P53 was enhanced after CD treatment in the nucleus **(Figure 6G and shown below)**. This result further indicated that endogenous P300 and P53 competitively combined with HIF-1α to regulate HMOX1 expression affecting ferroptosis of HASMCs.

**Q4).** **The expression "obviously" is used throughout the manuscript, nevertheless should be replaced because this is an original contribution no outcome is obvious, instead expected outcome or supports the hypothesis, etc.**

**Re:** Thank the reviewer for pointing out this problem, and we are sorry about it. We have revised these inappropriate descriptions in our manuscript.
